# Recognition of Facial Emotional Expressions Among Italian Pre-adolescents, and Their Affective Reactions

**DOI:** 10.3389/fpsyg.2018.01303

**Published:** 2018-08-03

**Authors:** Giacomo Mancini, Roberta Biolcati, Sergio Agnoli, Federica Andrei, Elena Trombini

**Affiliations:** ^1^Department of Education, Alma Mater Studiorum University of Bologna, Bologna, Italy; ^2^Marconi Institute for Creativity, Alma Mater Studiorum University of Bologna, Sasso Marconi, Italy; ^3^Department of Psychology, Alma Mater Studiorum University of Bologna, Bologna, Italy

**Keywords:** emotional recognition, facial expression, affective reactions to facial emotional expressions, emotional development, preadolescence

## Abstract

The recognition of emotional facial expressions is a central aspect for an effective interpersonal communication. This study aims to investigate whether changes occur in emotion recognition ability and in the affective reactions (self-assessed by participants through valence and arousal ratings) associated with the viewing of basic facial expressions during preadolescence (*n* = 396, 206 girls, aged 11–14 years, Mage = 12.73, *DS* = 0.91). Our results confirmed that happiness is the best recognized emotion during preadolescence. However, a significant decrease in recognition accuracy across age emerged for fear expressions. Moreover, participants' affective reactions elicited by the vision of happy facial expressions resulted to be the most pleasant and arousing compared to the other emotional expressions. On the contrary, the viewing of sadness was associated with the most negative affective reactions. Our results also revealed a developmental change in participants' affective reactions to the stimuli. Implications are discussed by taking into account the role of emotion recognition as one of the main factors involved in emotional development.

## Introduction

Facial expression recognition is an essential ability for good interpersonal relations (Niedenthal and Brauer, 2012), and a major subject of study in the fields of human development, psychological well-being, and social adjustment. In fact, emotion recognition plays a pivotal role in the experience of empathy (Gery et al., 2009), in the prediction of prosocial behavior (Marsh et al., 2007), and in the ability model of emotional intelligence (Salovey and Mayer, 1990). Additionally, the literature demonstrates that impairments in emotional expression recognition are associated with several negative consequences, such as difficulties in identifying, differentiating, and describing feelings. For example, several studies have shown an association of deficits in emotional facial expression processing with psychiatric disorders in both adults (Phillips et al., 2003) and children (Blair, 2003), alexithymia (Grynberg et al., 2012), and difficulties in social functioning (Batty and Taylor, 2006).

Numerous studies have identified six basic universally recognized emotions: anger, disgust, fear, happiness, sadness, and surprise (see, for instance, Ekman, 1992). The ability to recognize basic emotions emerges very early in life, as infants use emotional expressions as behavioral cues. In fact, from the first year of life, infants recognize emotions from faces and, therefore, can adjust their behavior accordingly during social interactions with a caregiver (Hertenstein and Campos, 2004). A more accurate understanding of emotions appears in pre-school age children (Widen and Russell, 2008), although it may be that 3-year-olds' facial expression recognition skills partly depend on task specifications. For example, Székely et al. (2011) found different recognition rates between verbal (emotion-labeling) and nonverbal (emotion-matching) tasks in the case of four basic emotions (happiness, sadness, anger, and fear), among normally developing 3-year-olds. Despite this, a progressive improvement occurs during school age through middle childhood, up to the emergence of mature recognition patterns in adulthood (Herba et al., 2006; Widen, 2013). However, it seems that recognition ability has different developmental patterns among emotions (Durand et al., 2007). For example, while happy and sad expressions seem to be accurately categorized early in life (Gosselin, 1995), there is no clear evidence regarding expressions of anger and disgust. Despite some inconsistencies, face processing abilities generally increase across childhood and adolescence (Ewing et al., 2017), and research generally agrees on the improvement in recognition performance with age (Theurel et al., 2016). Specifically, some studies have suggested that near-adult levels of recognition are achieved before adolescence (Rodger et al., 2015). However, it is worth mentioning that developmental researchers have used several methods to measure how accurately children recognize facial expressions of different emotions (Bruce et al., 2000), such as the discrimination paradigm, the matching procedure and free labeling. Hence, the ability to recognize most emotional expressions appears, at least partly, to be dependent on task demands. Additionally, while recognition of, and reactions to, facial expressions have been largely examined among children and adults in both natural and clinical settings, fewer studies have focused on preadolescence, providing mixed and inconsistent results. For example, Lawrence et al. (2015) tested the hypothesis that an individual's ability to recognize simple emotions through childhood and adolescence is modulated by pubertal stage. In contrast, Motta-Mena and Scherf (2017) affirmed that while the ability to process basic expressions improves with age, puberty, *per se*, does not specifically contribute in this sense. Indeed, once age is accounted for, there is no additional influence of pubertal development on the ability to perceive basic expressions in early adolescence.

In addition, to understand the mechanisms involved in recognition of facial expressions, two primary dimensions of emotional perception of faces have been identified (Russell, 1980; Schubert, 1999): valence and arousal. Valence represents the pleasantness of a face, and is measured on a linear scale with pleasant/positive emotions at one end, and unpleasant/negative emotions at the other. Arousal indicates the degree to which a face brings an observer to a state of greater alertness. Arousal and valence have long been studied, finding broad similarities between adults and children (Vesker et al., 2017). However, to our knowledge, there is a paucity of research examining preadolescents' affective ratings of facial expressions elicited by experimental stimuli. According to Lang et al. (1997), the variance in facial emotional assessments is accounted for by three major affective dimensions: pleasure, arousal, and dominance. In this study, we are particularly interested in affective valence (ranging from pleasant to unpleasant) and arousal (ranging from calm to excited). Affective reactions to facial emotional expressions in prepubescent boys and girls were examined, and compared to those of adults, by McManis et al. (2001). These authors demonstrated that children's and adolescents' affective evaluations of pictures, in terms of pleasure, arousal, and dominance, were similar to those of adults. However, more studies specifically focused on preadolescent youth are needed.

For the aforementioned reasons, the present study combined the categorical (ability) and dimensional (valence and arousal) approaches to explore recognition ability and affective ratings of valence and arousal in preadolescence, which is considered the phase of development ranging between 11 and 14 years of age (Meschke et al., 2012). Specifically, the primary purpose of the present study was to examine the ability to recognize basic emotions from a standardized set of emotional facial expressions in a sample of Italian preadolescents. It seems logical to hypothesize a change in such an ability during preadolescence, when pupils move on to secondary school and significantly improve their social relations with peers and thereby their ability to process basic facial expressions of emotion. For this reason, we expect differences in emotion recognition trajectories between expressions. We also expect that, overall, some facial expressions that are perceived as simpler, such as happiness, will be more easily recognized than others, as has been found in some previous research (e.g., Gagnon et al., 2014).

The secondary aim was to investigate age and gender differences in the affective reactions elicited by facial emotional expressions, in terms of valence and arousal. In particular, we assume that girls and boys have similar affective reactions to facial emotional stimuli for the explored expressions. Moreover, we expect that the arousal effects of viewing facial expressions will decrease significantly with increasing age. Preadolescents, through constant exposure to emotional facial expressions and the gradual acquisition of social display rules, could gradually get used to the affective states associated with the vision of these emotional stimuli, moving to patterns close to those of adults (Vesker et al., 2017).

The results will be compared with findings from a previous study that explored emotion recognition in late childhood by means of the same experimental procedure (Mancini et al., 2013). As evident differences in results on the developmental trajectories of the recognition of specific emotions have emerged from the literature, due to the variability across studies in task demands and/or in the type of response required from participants, we made this comparison possible by adopting the same stimuli and procedures described in the previous study.

## Materials and methods

### Participants

The sample included 396 preadolescents (206 females) ranging in age from 11 to 14 years (mean age 12.73 years; *SD* = 0.91). Participants were selected from three different State-run middle schools in central and northern Italy. The sample consisted of 134 (33.9%) participants (47% females) in the first grade, 120 (30.4%) participants (58.3% females) in the second grade, and 141 (35.7%) participants (52.5% females) in the third grade. Students were excluded if they reported a diagnosis of intellectual or psychological disabilities certified by the public mental health service.

### Stimuli and apparatus

Forty-eight color pictures of faces representing five basic emotions (anger, fear, sadness, happiness, and disgust) and neutral expressions were selected from the Karolinska Directed Emotional Face System (Lundqvist et al., 1998). Posing subjects were amateur actors of both sexes, aged between 20 and 30 years old. The selection criteria were: no beards, mustaches, earrings or eyeglasses, and preferably no visible make-up during the photo-session. Eight faces among younger actors were selected for each emotional expression, with both male and female presenters included for each expression (half of the faces selected were male and half were female). The 48 pictures were divided into two sets of 24 facial expressions, and each set was arranged in four blocks of six expressions, such that there was one exemplar from each of the six stimulus types in each block. For each set, four different orders of picture presentation were constructed. Stimulus presentation was conducted using an Acer laptop computer with a 2.4 GHz processor and a 21-inch monitor. A refresh rate of 60 Hz and a resolution of 1,440 × 900 pixels were used.

Participants were asked to complete the facial expression recognition task (selecting one of six emotion labels), and to rate their valence and arousal on a 9-point scale using the paper-and-pencil version of the Self-Assessment Manikin (SAM; Bradley and Lang, 1994), which measured the pleasure and arousal associated with viewing each picture. The unlabeled dimensions of pleasure and arousal were represented pictorially by a SAM figure. The pleasure scale showed the SAM smiling, happy at one extreme and unhappy at the other. Arousal was represented by a relaxed, sleeping figure at the calm end of the scale and a jumping, excited and wide-eyed figure at the other.

### Design and procedure

The study was approved by the Ethical Committee of the Department of Psychology and Educational Science of the University of Bologna. The experimental procedure was illustrated to the teachers, students and their parents in a presentation session. Parents gave their written consent and the children were freely allowed to participate in, or abstain from, the research.

Participants were tested individually in a quiet room that was arranged for the experimental procedure. To assure a correct understanding of the task, the procedure was explained first to all participants in their classrooms and then once again to each participant individually by means of the presentation of two pictures. Participants were tested in a randomized order. They sat approximately one meter from the computer screen on which the pictures were presented. Each face was presented on the screen for a 6-s interval, and after each picture offset, participants were asked to complete a facial expression recognition task and to rate their valence and arousal. The rating period was a total of 20 s (10 s to label the expression and 10 s for arousal and valence ratings), allowing ample time for scores. A 30-s interval lapsed between the presentations of each picture. The order in which pictures were viewed was varied in eight different ways across participants.

To complete the emotion recognition task, participants were asked to select one of six emotion labels (i.e., anger, sadness, happiness, fear, disgust, or neutral) that best described the emotional expression they had just seen. The labels were shown on an answer sheet, and participants made their responses by drawing a cross on the emotion label. The order of the labels varied from item to item and was randomized for each participant, to reduce the tendency to select some labels more than others simply because of primacy.

Participants were given 10 s to make their selection, and they were asked to respond as accurately as possible. No feedback was given regarding the appropriateness of any response. Responses were coded offline as accurate or inaccurate using a dichotomous variable. An average recognition accuracy score was then obtained for each emotion.

After the emotion recognition task, participants were also required to rate their affective responses to facial emotional expressions viewed via the SAM system. Participants were asked how they felt after seeing each facial expression: specifically, “How pleasant was your reaction when looking at the face?” and “How excited did you feel when looking at the face?” for the valence and arousal dimensions, respectively. The pleasure dimension was presented first, and participants were instructed to give their responses on the page for each dimension, within 10 s. A mark could be made on or between the figures.

### Data analysis

Recognition accuracy and affective reactions elicited by facial emotional expressions, measured through valence and arousal, were explored in three separate generalized linear mixed models and treated as repeated dependent variables (Toplitz covariance structure). Robust error estimation was used to control for the effect of outliers (Wu, 2009). Emotional expression (anger, sadness, happiness, fear, disgust, neutral face) was entered in the models as a categorical within-subjects effect, gender (male, female) was entered as a categorical between-subjects effect, whereas age (from 133 to 177 months) was entered as a continuous covariate effect. Two-way interactions between the previous variables were added to the models. Finally, because of the possible nested nature of our data (students nested within schools), school was added to the models as a nested factor to control for this.

## Results

### Emotion recognition accuracy

A main effect of emotional expression emerged from the first linear mixed model predicting recognition accuracy, *F*_(5, 790.51)_ = 4.511, *p* < 0.001. As illustrated in Table 1, happy expressions were the most recognized emotions (*p*s < 0.004), followed by anger, disgust and neutral expressions, while sadness and fear were significantly less recognized than all the other emotions (*p*s < 0.001). Neither an effect of gender, *F*_(1, 394.99)_ = 0.364, *p* = 0.546, nor an interaction effect between gender and emotional expression, *F*_(5, 789.664)_ = 1.091, *p* = 0.364, emerged from the model.

**Table 1:** Means and standard deviations of emotions as a function of the facial expression presented.

**Facial expression presented**	Accuracy %^*^	
	***M***	***SD***
Anger	94.21	14.58
Disgust	92.90	16.59
Fear	85.49	19.80
Happiness	98.12	9.20
Sadness	86.46	22.56
Neutral	92.34	16.29

* *Percentage range 0–100*.

Finally, a statistically significant interaction occurred between emotional expression and age, *F*_(5, 790.480)_ = 4.730, *p* < 0.001. In particular, a statistically significant decrease in recognition accuracy across months emerged for fear expressions, *b* = −0.219, *t*_(1036.17)_ = −3.316, *p* = 0.001, 95% CI [−0.205, −0.005].

### Affective reactions: valence

Means and standard deviations were calculated for valence and arousal elicited by each emotion and neutral expression (as shown in Table 2).

**Table 2:** Means and standard deviations of valence and arousal.

**Presented facial expression**	Valence^*^		Arousal^*^	
	***M***	***SD***	***M***	***SD***
Anger	4.15	1.62	4.42	1.80
Disgust	4.24	1.65	4.03	1.75
Fear	4.51	1.51	4.68	1.78
Happiness	6.92	1.61	5.35	1.95
Sadness	3.47	1.42	3.91	1.72
Neutral	4.24	1.23	2.79	1.43

* *rated on 9-point scale. The valence ratings ranged from 1 = very unpleasant to 9 = very pleasant. The arousal ratings ranged from 1 = calm to 9 = excited*.

**Figure 1:**
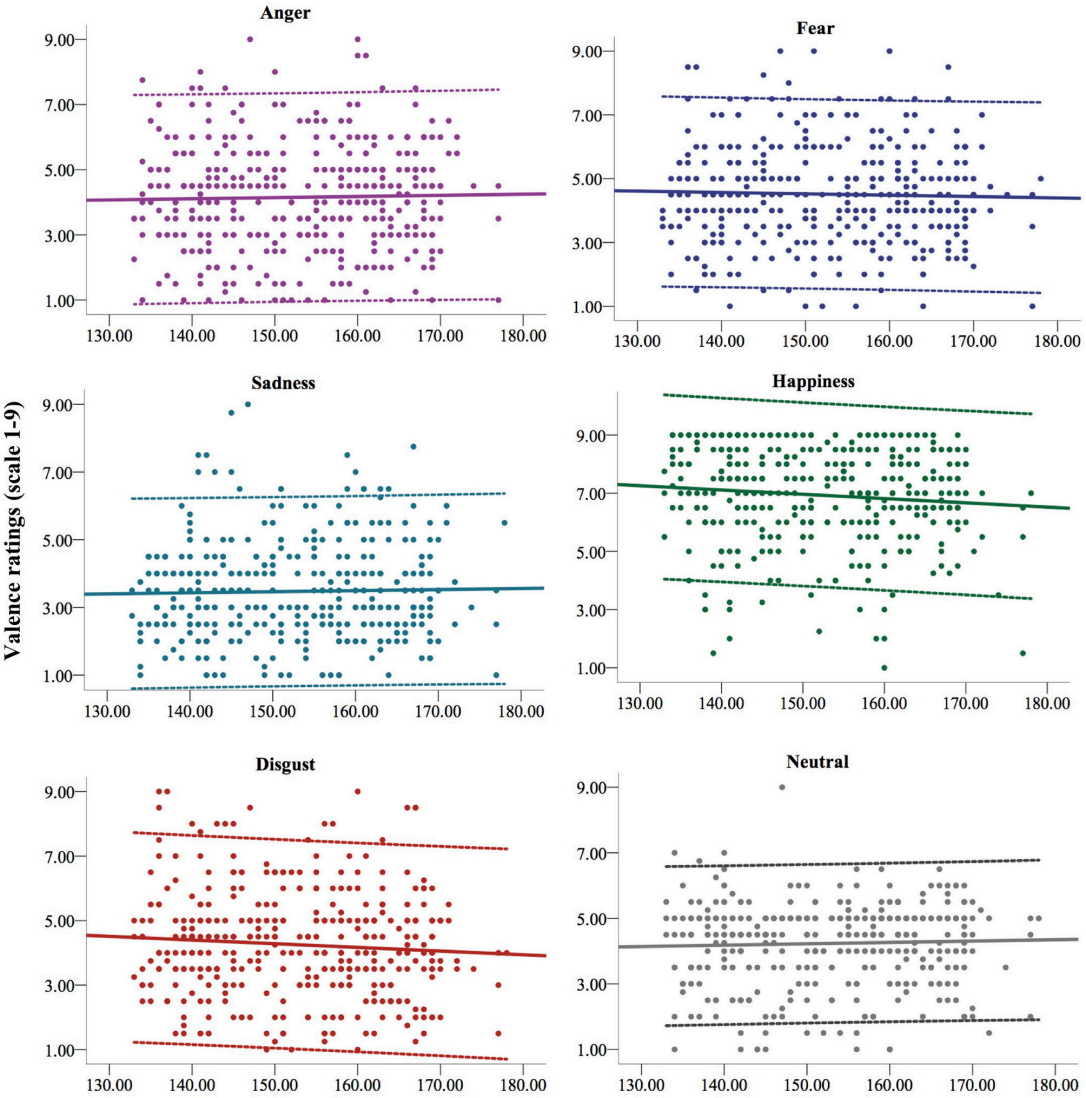
The figure depicts the changes in the valence of the affective reactions to the vision of the five basic facial expressions of emotion and the neutral face from 11 (133 months) to 14 years (177 months) of age. Continuous lines represent the linear regression lines, dotted lines represent upper and lower confidence intervals (95% CI).

A first statistically significant main effect of emotional expression emerged from the linear mixed model predicting valence, *F*_(5, 891.03)_ = 7.156, *p* < 0.001. As expected, happy facial expressions elicited more pleasant reactions compared to all the other emotional expressions and to neutral expressions (*p*s < 0.001). Among unpleasant emotions, facial expressions of sadness elicited more unpleasant reactions (*p*s < 0.001) compared to the other emotional expressions and to neutral expressions. A significant interaction between gender and emotional expression emerged from the model, *F*_(5, 892.08)_ = 5.482, *p* < 0.001, showing in particular that girls had more positive reactions than boys to viewing happy expressions, *b* = 0.667, *t*_(1205.69)_ = −3.707, *p* < 0.001, 95% CI [0.314, 1.020]. Finally, a significant interaction between age and emotional expression emerged from the analysis, *F*_(5, 891.14)_ = 2.455, *p* = 0.032. As depicted in Figure 1, this effect specifically showed a decrease in positive reactions to the sight of happy facial expressions with increasing age, *b* = −0.019, *t*_(1203.84)_ = −2.441, *p* = 0.015, 95% CI [−0.035, −0.003] and an almost significant decrease in positivity with increasing age to the sight of expressions of disgust, *b* = −0.015, *t*_(1282.50)_ = −1.722, *p* = 0.077, 95% CI [−0.032, 0.001].

### Affective reactions: arousal

**Figure 2:**
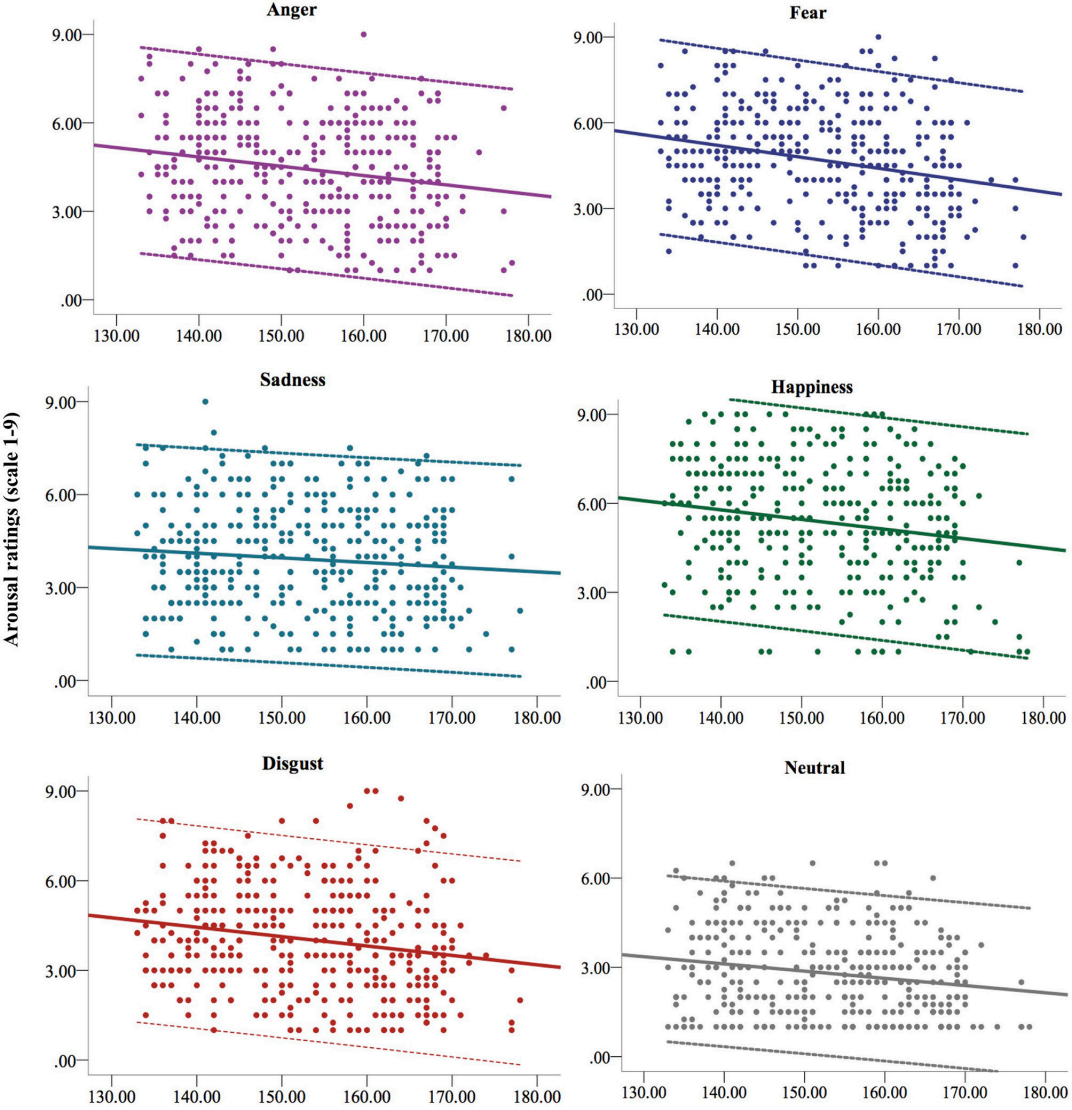
The figure depicts the changes in the arousal of the affective reactions to the vision of the five basic facial expressions of emotion and the neutral face from 11 (133 months) to 14 years (177 months) of age. Continuous lines represent the linear regression lines, dotted lines represent upper and lower confidence intervals (95% CI).

A statistically significant main effect of emotional expression emerged from the linear mixed model predicting arousal, *F*_(5, 902.86)_ = 3.926, *p* = 0.002. Facial expressions of happiness elicited significantly more aroused reactions (*p*s < 0.001), whereas neutral expressions elicited less aroused reactions (*p*s < 0.001) than all the other emotional expressions. A significant interaction between gender and emotional expression emerged from the model, *F*_(5, 896.58)_ = 6.755, *p* < 0.001, showing in particular that girls had more aroused reactions than boys to viewing happy facial expressions, *b* = 0.711, *t*_(1271.37)_ = −3.657, *p* < 0.001, 95% CI [0.329, 1.093]. Finally, a significant main effect of age emerged from the model, *F*_(1, 395.81)_ = 32.37, *p* < 0.001. As depicted in Figure 2, this effect showed a generalized decrease in arousal with increasing age when viewing emotional and neutral expressions.

## Discussion

Our study investigated the emotion recognition accuracy and the perception of arousal and valence ratings of facial expressions among preadolescents. The findings showed that some changes in facial expression recognition occur during preadolescence. Specifically, recognition accuracy varies according to emotions and the observer's age. Moreover, our results highlight that the affective ratings in response to emotional facial expressions are also subjected to a developmental process during this stage of life.

### Emotion recognition accuracy

In general, preadolescents aged 11–14 years were highly successful at emotion recognition. The scores for emotion recognition were generally quite high, with over 85% accuracy for all the basic emotions; it is noteworthy that accuracy for recognition of happy, angry, disgusted and neutral faces scored over 92%.

In a previous study (Mancini et al., 2013) adopting the same experimental procedure with children aged 8–11, accuracy was lower for almost all emotions except for happiness and anger, which showed very similar recognition patterns. This discrepancy in results might suggest that, in general, accuracy in emotional recognition improves during the transition from late childhood to preadolescence (Theurel et al., 2016). However, similar recognition trends emerged from both studies.

The small age-related changes in overall accuracy rates for basic emotions are not surprising. Indeed, as suggested by Motta-Mena and Scherf (2017), pubertal development shapes sensitivity to perception of complex social expressions, but not basic ones.

In preadolescence, findings regarding emotion recognition accuracy show that happy expressions are the best recognized emotions, followed by angry, disgusted and neutral expressions, while sad and fearful expressions are significantly less recognized as compared to all the other emotions. Our findings are consistent with several studies showing that happiness continues to be the most recognized emotion during development (Vicari et al., 2000; Herba et al., 2008). In contrast, which emotion is the least recognized is a controversial issue. Some studies (Boyatzis et al., 1993; Herba et al., 2006) found that anger was the most difficult to recognize, whereas others have reported sadness as the least recognized (Philippot and Feldman, 1990; Holder and Kirkpatrick, 1991; Lenti et al., 1999; Chronaki et al., 2013, 2015). Our results are consistent with those of the extant research, suggesting that sadness is one of the least accurately recognized expressions of emotion and that, akin to late children (Mancini et al., 2013), preadolescents are less accurate in decoding sadness and fear.

Generally, scholars (Theurel et al., 2016) have observed that the recognition rate for basic emotions increases significantly between 5 and 15 years of age. However, our results contrast with this general assumption. Indeed, by analyzing the development of emotional facial expression recognition accuracy during preadolescence, a statistically significant decrease in recognition accuracy across months emerged in the analysis for fear.

The combined findings emerging from the present study and from a previous study (Mancini et al., 2013) therefore suggest that the developmental pattern is not uniform across all basic emotions (Durand et al., 2007), but different developmental trajectories characterize the recognition of specific expressions of basic emotions. Note that the decrease in accuracy observed may also be due to the older participants devoting less care to the task.

Moreover, preadolescents aged 11–14 were highly successful at neutral expression recognition. Previous studies have described neutral faces as emotionally ambiguous to children and to adults (Lee et al., 2008) and have often been misinterpreted as negative expressions (Waters et al., 2010). These data are not confirmed by the results emerging from the present study. The enhancement of neutral face recognition found in the present work and in the previous research targeting late childhood (Mancini et al., 2013) suggests that late childhood, but in particular preadolescence, could be considered a critical period for disambiguating the meaning of this facial expression. Another possible explanation for this finding is linked to the methods used to index facial emotion recognition: In the Karolinska set the emotional faces are quite intense and thus a neutral face may be quite easy to discriminate from the others.

As for sex differences, our study showed no evidence among preadolescents in facial expression recognition, as suggested in a previous work by Montirosso et al. (2010). Our results suggest that during preadolescence boys might be able to fill the gap that in childhood had been registered between males and females (McClure, 2000). Our results, for example, showed that from an initial disadvantage for sadness recognition, boys became as accurate as girls after 11 years of age (Mancini et al., 2013). Previous studies have shown that the developmental trend for face emotional recognition in childhood varies according to gender-specific maturation of some brain areas involved in processing negative emotions (Tremblay et al., 2001; Herba and Phillips, 2004; Thomas et al., 2007) or to sex differences determined by the adult-guided interaction influencing early childhood. Girls develop initial emotion recognition abilities more rapidly than boys (McClure, 2000) because they are exposed to a more expressive environment (Fivush, 1991). However, during late childhood, the gap between males and females tends to reduce progressively until it disappears. In line with Mancini et al. (2013), our results demonstrated that sex differences in facial expression recognition are transient and unstable during development.

### Affective response

Our study provided new findings regarding affective ratings of valence and arousal to emotional facial expressions during preadolescence. The present work represents a preliminary exploration of this issue during this stage of life.

Among unpleasant emotions, the affective reactions of preadolescents to facial expressions of fear and sadness were less unpleasant and more unpleasant, respectively, compared to the other emotional expressions and to neutral expressions. The vision of facial expressions of happiness elicited more arousing affective reactions, whereas the vision of neutral expressions elicited less arousing affective reactions as compared to vision of the other emotional expressions. Facial expressions of happiness elicited a more pleasant affective response to facial emotional expressions than all other expressions in preadolescents. As expected, facial expressions of happiness were judged as more pleasant as compared to all the other emotional expressions and to neutral expressions. Facial expressions of happiness elicited more arousing reactions than all of the other emotional expressions and neutral expressions. Sadness yielded the most unpleasant reactions, followed by anger, neutral expressions, disgust, and fear. Neutral facial expressions elicited the least arousing reactions, followed by facial expressions of sadness, anger, disgust and fear. Our findings are consistent with the results of the previous study on late childhood participants (Mancini et al., 2013) and with a classical trend in the arousal reaction elicited by emotional faces that emerged in adult studies (Gerber et al., 2008; Goeleven et al., 2008).

As far as gender differences are concerned, girls reported higher affective reactions to happy facial expressions than boys in terms of valence and arousal ratings. This result could be determined by the typical female attitude toward positive stimuli connected with pro-social behavior. Moreover, girls were more aroused than boys from the vision of fear expressions. As suggested by studies on gender and emotion (Brody and Hall, 2008; McCormick et al., 2016) it could be that females more accurately display gender-stereotypic expressions, that is they can express fear and happiness more accurately.

It is worth noting that preadolescents' valence and arousal ratings seem to be lower compared to the values that emerged in the study with the child population (Mancini et al., 2013). Specifically, the ratings emerging in the present study are comparable with the valence and arousal appraisals emerging in adolescents and adults, but specific differences in the preadolescent sample were found. Our results showed that the affective space associated with the viewing of facial expressions is subjected to a typical developmental process during preadolescence. Indeed, our findings revealed a significant decrease in the valence reported after viewing happy expressions and an almost significant one for disgusted expressions. In particular, the results showed that valence ratings reported after the sight of both expressions became more negative during months. This negative trend could be interpreted as the consequence of a better comprehension of these affective dimensions, inasmuch as it is more similar to adults' evaluations.

Moreover, the self-reported arousal elicited by faces was characterized by a significantly negative trend, and the activation determined by the viewing of emotional expressions showed a decrease over time for all the emotions. In late childhood, the same decremental trend involved only the happiness and neutral expressions. Our results confirmed that the subjective affective space, and in particular the activation associated with emotional expressions, is subject to an accommodation process starting in late childhood and consolidating in preadolescence. The affective reactions elicited by facial emotional expressions, as measured through self-report ratings, assume a more mature aspect, typical of the adult period. In particular, the decrease in arousal emerging during preadolescence is consistent with data regarding adults' arousal ratings in response to affective faces. However, the observed negative trends for arousal could be interpreted as the result of the preadolescent's acquisition of greater expertise and comprehension of the emotional dimensions underlying facial expressions. Both categorical and dimensional accounts of emotion perception therefore seem to highlight a developmental trend during preadolescence that takes emotional perception to a more mature form.

## Conclusions

This research, which aimed to contribute to the literature on the ability to recognize and to rate arousal and valence of facial expressions among preadolescents, has several limitations. The study used a cross-sectional design and findings were compared to the results obtained from a previous study on late children; in future studies it would be beneficial if the models were examined using a longitudinal approach.

Another limitation could be related to the nature of stimulation used in the present study. Our stimuli were limited to photographs of young adults. However, given some evidence that peer faces may be especially salient to pre-adolescents and adolescents, it would be useful to employ them in a future research task to determine whether this can change the performance of children and preadolescents.

Moreover, stimuli in the present study were presented for a relatively long duration (6 s) and this could increase the performance accuracy and make the task less prone to discrimination among participants. In addition, the disappearance of the stimulus from the screen after 6 s might involve the activation of short-term memory, which was not investigated. Nevertheless, in the present study we carefully included the same facial expressions from the stimulus set of Mancini et al. (2013) in order to make possible the comparison between the two studies. Indeed, the different methodologies and age groups used in the literature, together with the differing emotions included, is one of the main limitations that make it difficult to comprehensively understand the quantitative and qualitative developments in emotion recognition during this period of life (Lawrence et al., 2015).

The current work has demonstrated specific changes in emotional recognition, as well as in affective ratings to emotional faces, with the increase in the participants' ages. This is one of the first studies to explore these issues in preadolescence, and the first one to show an age effect in both emotional face recognition accuracy and in affective reactions elicited by facial emotional expressions. We hope, therefore, that the findings emerging in the present work will be replicated and further explored in future studies. In particular, it is worth emphasizing the importance of understanding which elements can contribute to the emerging age effect. Ad hoc analyses exploring variables that are usually associated with age changes during preadolescence (e.g., cognitive abilities, nervous system, social abilities, empathy, etc.) might especially contribute to the development of appropriate statistical models to predict the changes in emotional recognition accuracy and affective ratings to facial emotional expressions.

This would also allow us to contribute to the clinical research on preadolescence. Identifying preteens with difficulties in recognizing other people's emotions and in expressing their feelings would enable the adoption of precocious interventions in the promotion of well-being in relation to emotional competencies. The importance of acting preventatively in early adolescence is due to the fact that in this phase of life some personality vulnerabilities are associated with several at-risk behaviors (Biolcati et al., 2016). This could be realized in schools or in educational group contexts in order to develop those protective factors that can adjust a risky path or help to strengthen a good adjustment (Sameroff, 2006). Despite the importance of emotional development for well-being throughout life, the development of emotional skills over the preadolescence period remains surprisingly under-examined. Starting from this lack of literature, we have tested the trend of emotional recognition ability and of affective ratings to emotional facial expressions in a large sample of middle school students. Our results suggest that preadolescence is an age where the evolutionary trajectory of both emotion recognition and affective reactions to faces does not proceed in a linear way, as happens during childhood, but undergoes some changes probably due to the typical adjustments of this development stage.

## Author contributions

GM conceived of the presented idea, carried out the experiment and wrote the manuscript. RB helped to develop the theoretical framework and wrote the manuscript with support from GM. SA contributed to sample preparation, performed the statistical analyses and wrote the Results section with support from GM. FA helped supervise the project and contributed to the interpretation of the results. ET supervised the findings of this work. All authors discussed the results and contributed to the final manuscript.

### Conflict of interest statement

The authors declare that the research was conducted in the absence of any commercial or financial relationships that could be construed as a potential conflict of interest.
